# New Appliances and Things Medical

**Published:** 1899-03-25

**Authors:** 


### NEW APPLIANCES AND THINGS MEDICAL.

[We shall be glad to receive, at our Office, 28 & 29, Southampton Street, Strand, London, W.C., from the manufacturers, specimens of all new preparations and appliances which may be brought out from time to time.]

### EUCALYPTUS OIL (BYARD'S).

(Langton, Fort, and Co., 20, St. Dunstan's Hill, E.C.)

We have before us a sample of Byard's pure volatile oil. It is stated to be distilled from the Eucalyptus maculata (var. citriodora) at Gladstone, Queensland. Those who are familiar with the odour attaching to the ordinary crude Eucalyptus oil, or even the more refined varieties commonly met with, will hardly recognise the deliciously fragrant aroma of Byard's preparation as one and the same class. For internal and external use Eucalyptus oil of such agreeable qualities is sure to find many admirers.

### PROTENE SPECIALITIES (BISCUITS, BREAD, &c.)

(The Protene Company, Limited, 36, Welbeck Street, W.)

A variety of biscuits made by a special process by the above firm have been submitted to us for examination. The basis of all these specialities is a particular kind of "flour," called protene, from the fact that it consists almost entirely of proteid. It is prepared from the casein of milk, and is chemically of the same nature as, and has the same nutritive properties as, the proteid of meat, eggs, and flour. That a flour free from farinaceous admixture and of so high a nutritive value can be made the basis of such a variety of foods should be welcomed as a boon by those who cannot tolerate a farinaceous diet. For the diabetic, the rheumatic, and obese patient such an opportunity should be of special value, for by it they are enabled to enjoy many of the good things of this life which without it would have to remain forbidden fruits. For instance, of biscuits and cakes there are among other varieties cheese, vanilla, salt, almond, chocolate, luncheon, training, sponge cakes, &c., and where all form of carbohydrate is contra-indicated there are protene biscuits sweetened with saccharine. The protene flour can be made up into bread puddings and jellies, and in these directions is likely to become a household desideratum. We have eaten several of the biscuits, and from a gastronomic point of view they are as satisfactory as from a standpoint of nutritive and therapeutic value.

### ARTIFICIAL HAIR.

(Sanitary Mattress Co., 11, London Street, Fenchurch Street, and 347, Cable Street, London, E.)

The sample of artificial hair which has been forwarded us by the above company is indeed a triumph of modern ingenuity. Superficially it closely resembles horsehair of the finest quality, it is perfectly odourless, and from a sanitary point of view it has the advantage of having been subjected to process which renders it completely sterile. There is a fine elasticity and spring about the material, and the mattresses and cushions into which it is converted offer obvious and solid advantages. The prices quoted by the firm should be a sufficient inducement for hospitals and institutions to give this new departure a trial. Sample mattresses can be seen at the offices, 11, London Street, Fenchurch Street, E.C.

